# Differences in Short QT Syndrome Subtypes: A Systematic Literature Review and Pooled Analysis

**DOI:** 10.3389/fgene.2019.01312

**Published:** 2020-01-17

**Authors:** Laura S. Raschwitz, Ibrahim El-Battrawy, Kim Schlentrich, Johanna Besler, Michael Veith, Gretje Roterberg, Volker Liebe, Rainer Schimpf, Siegfried Lang, Christian Wolpert, Xiaobo Zhou, Ibrahim Akin, Martin Borggrefe

**Affiliations:** ^1^ First Department of Medicine, Faculty of Medicine, University Medical Centre Mannheim (UMM), University of Heidelberg, Mannheim, Germany; ^2^ DZHK (German Centre for Cardiovascular Research) Partner Site Heidelberg/Mannheim, Mannheim, Germany

**Keywords:** short QT syndrome, sudden cardiac death, channelopathy, outcome, cardiac arrest

## Abstract

**Background:**

Short QT syndrome (SQTS) is a rare syndrome and affects different types of genes. However, data on differences of clinical profile and outcome of different SQTS types are sparse.

**Methods:**

We conducted a pooled analysis of 110 SQTS patients. Patients have been diagnosed between 2000 and 2017 at our institution (n = 12) and revealed using a literature review (n = 98). 29 studies were identified by analysing systematic data bases (PubMed, Web of Science, Cochrane Libary, Cinahl).

**Results:**

67 patients with genotype positive SQTS origin and 43 patients with genotype negative origin were found. A significant difference is documented between the sex with a higher predominance of male in genotype negative SQTS patients and predominance of females in genotype positive SQTS patients (male 52% versus 84%, female 45% versus 14%; p = 0.0016). No relevant difference of their median age (genotype positive 27 ± 19 versus genotype negative 29 ± 15; p = 0.48) was found. Asymptomatic patients and patients reporting symptoms such as syncope, sudden cardiac death, atrial flutter and ventricular fibrillation documented in both groups were similar except atrial fibrillation (genotype positive 19% versus genotype negative 0%; p = 0.0055). The QTc interval was not significantly different in both groups (genotype positive 315 ± 32 versus genotype negative 320 ± 19; p = 0.30). The treatments (medical treatment and ICD implantation) in both groups were comparable. Electrophysiology studies were not significantly higher documented in patients with genotype positive and negative origin (24% versus 9%; p = 0.075). Events at follow up such as VT, VF, and SCD were not higher presented in patients with genotype positive (13% versus 9%) (p = 0.25). 54% of genotype positive SQTS patients showed SQTS 1 followed by STQS 2 (21%) and SQTS 3 (10%).

**Conclusions:**

The long-term risk of a malignant arrhythmic event is not higher in patients with genotype positive. However, patients with genotype positive present themselves more often with AF with a female predominance. Also, other events at follow up such as syncope, atrial flutter and palpitation were not significantly higher (9% versus 0%; p = 0.079).

## Introduction

Short QT syndrome (SQTS) is an inherited channelopathy associated with a high risk of sudden cardiac death (SCD) (Gaita et al., 2003). Commonly found in all SQTS types is an abbreviated QTc interval. SQTS patients may present themselves with different symptoms such as atrial arrhythmias, palpitation and in worst cases SCD. Due to the high risk of SCD a family screening is mandatory to patients who have been diagnosed with SQTS (Giustetto et al., 2011; Priori et al., 2015). To prevent SCD implantable cardioverter defibrillation (ICD) is recommended (Gaita et al., 2003; Priori et al., 2015). On the other hand, a recently published study has shown that the implantations of ICD are associated with a higher risk of device-related complications. Therefore, an indication should be evaluated by a referral centre with good experience in SQTS (El-Battrawy et al., 2019). In addition, recently published data have shown that hydroquinidine prevent arrhythmias (Giustetto et al., 2011; Mazzanti et al., 2017). Other antiarrhythmics such as carvediol, metoprolol, and sotalol do not have the same effect as hydroquinidine (El-Battrawy et al., 2018a). However, hydroquinidine might not be effective in all SQTS types and its effect might be genotype dependent (McPate et al., 2005; McPate et al., 2008; Hu et al., 2017).

Since describing this rare arrhythmic disorder (Gussak et al., 2000) different SQTS types have been proposed such as SQTS 1 - 3 with a gain of potassium channels, SQTS 4 - 6 with a loss of calcium channels and a new SQTS with a cardiac Cl/HCO3 exchanger AE3 (Thorsen et al., 2017). However, the most frequent described type is SQTS 1. In some cases, overlap syndromes, like SQTS accompanied with Brugada syndrome (BrS), have been reported (McPate et al., 2005; Hu et al., 2017).

Comparison of clinical profile and the outcome between patients with genotype positive and patients with genotype negative are sparse. Therefore, we analyzed 29 studies including patients at our institution and conducted a comprehensive pooled analysis to present a clinical profile, diagnostic assessments, and outcome. In our study we define genotype negative SQTS patients as patients with negative testing. Patients with no testing at all are excluded in our study.

## Methods

The diagnosis of SQTS was based on the published Gollob criteria or on the published ESC criteria (Gollob et al., 2011; Priori et al., 2015). Secondary, a cascade family screening was targeted. In all, 29 studies were found with 27 families. Patients were followed regarding the risk of arrhythmic event such as syncope which was based on the general definition with transient loss of consciousness, and/or SCD. For measuring the QT interval, the tangent method in the precordial lead presenting the highest T-wave amplitude in V2 or V3 was used.

The study was agreed by the local Ethics Committee of the University hospital Mannheim.

### Systematic Literature Review

For the pooled analysis, publication dates up to December 2017 were included. Data bases^1^ were screened by a librarian using eligibility criteria such as English language and human subjects in order to include any study published up to December 2017.

Considering the clinical profile of patients, 626 studies were screened (JB, KS). Due to nonclinical studies, lack of information and overlapping data, 581 studies were excluded. Through systematic database analysis and their data analysed according to our model, 42 studies were identified. To identify significant literature for our clinical question we used the PICO strategy. For statistical analysis, the software SPSS version 25 (IBM, Tialy) was used.

**Figure 1:**
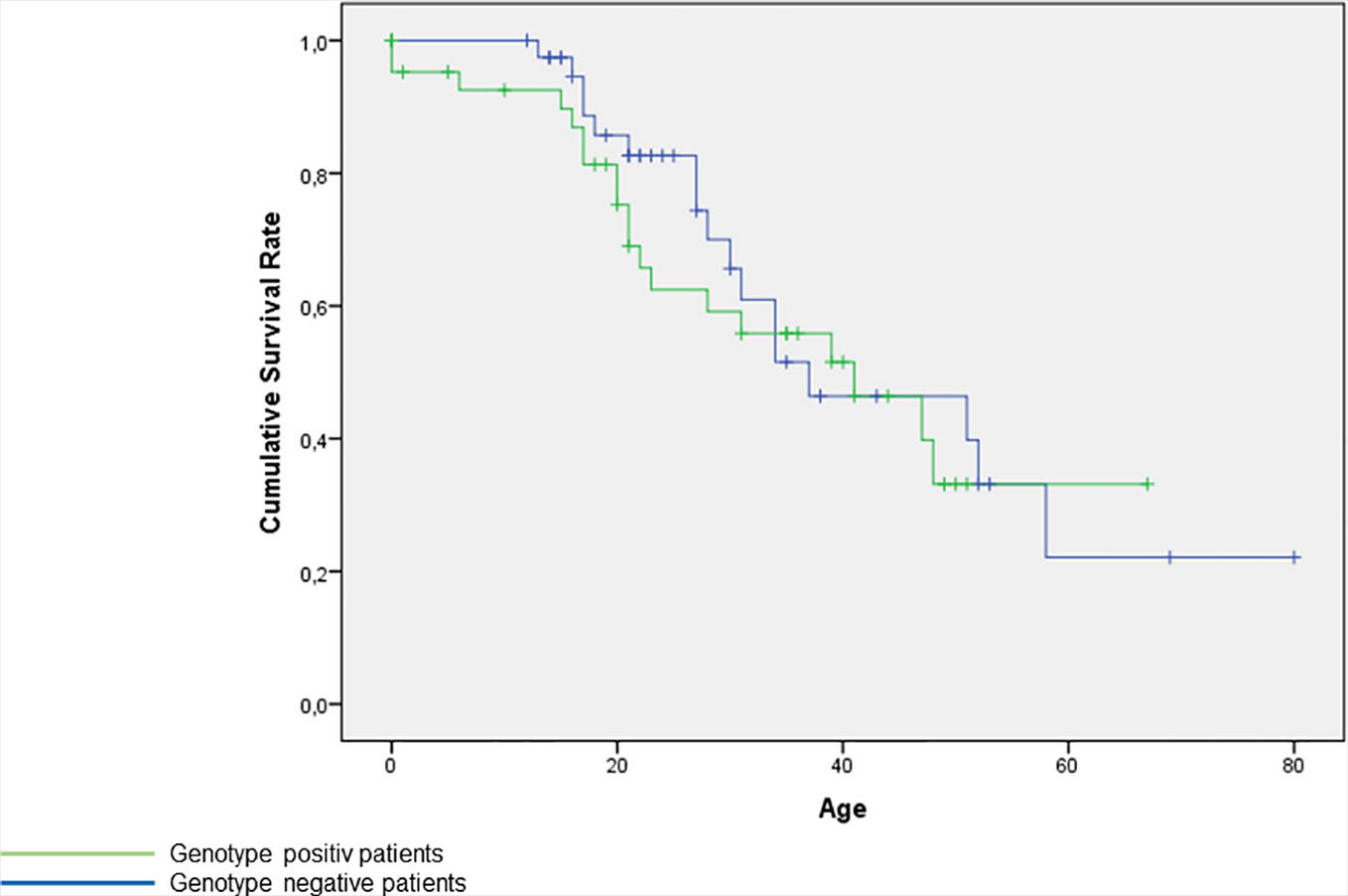
Flowchart showing the procedure to exclude irrelevant studies in the literature search.

The criterion SQTS was included for the studies selected in our analysis (**Figure 1**).

### Statistics

Continuous variables with a normal distribution are presented as mean ± SD, continuous variables with a non-normal distribution as median (interquartile range) and categorical variables as frequency (%). To assess normal distribution the Kolmogorov-Smirnov test was used. Student's t-test and the Mann-Whitney U-test were used to compare continuous variables with normal and non-normal distribution. Chi-squared-test or Fisher's exact test were used to compare categorical variables.

## Results

### Baseline Characteristics of SQTS Families

**Table S1** illustrates data collected from all studies. Having done a systematic literature review in addition, an analysis of a total of 110 patients from 29 studies were included (**Tables 1** and **2**).

**Table 1:** Overall comparison between confirmed gene mutation and no mutation found.

Overall N = 110	Confirmed gen N = 67	No mutation found N = 43	p-value
*Gender, n (%)*	Unknown = 2 (3)	Unknown = 1 (2)	0.6432
*Male*	35 (52)	36 (84)	0.0016
*Female*	30 (45)	6 (14)	0.0016
*Demographics*			
*Age, mean*	27 ± 19	29 ± 15	0.486
*Symptoms*			
*Syncope*	12 (18)	9 (21)	0.8850
*Palpitation*	9 (13)	1 (2)	0.0851
*Sudden cardiac death at admission*	12 (18)	14 (33)	0.1249
*Atrial flutter*	2 (3)	0 (0)	0.5194
*Atrial fibrillation*	13 (19)	0 (0)	0.0055
*nsVT*	2 (3)	0 (0)	0.5194
*asymptomatic*	25 (37)	19 (44)	0.6041
*ECG Data, mean (n)*			
*QTc (ms)*	315±32	320±19	0.302
*Medical treatment, n (%)*			
*Yes*	34 (51)	19 (44)	0.6338
*ICD-Implantation, n (%)*			
*yes*	23 (34)	15 (35)	0.9523
*EPS, n (%)*	16 (24)	4 (9)	0.0754
*Inducible arrhythmia (% from EPS)*	11 (16)	2 (5)	0.0747
*Follow-up time, mean (days)*	2823,87 ± 2106	1831,84 ± 1936	0.123
*Events at follow-up, n (%)*	15 (22)	4 (9)	0.1195
*nsVT/VT*	5 (7)	3 (7)	1.0000
*Aborted SCD (VF)*	3 (4)	0 (0)	0.5194
*Death*	1 (1)	1 (2)	1.0000
*Atrial fibrillation*	5 (7)	0 (0)	0.1543
*Syncope*	0 (0)	0 (0)	1.0000
*Atrial flutter*	0 (0)	0 (0)	1.0000
*Palpitation*	1 (1)	0 (0)	1.0000

nsVT, non-sustained ventricular tachycardia; VT, ventricular tachycardia; SCD, sudden cardiac death; VF, ventricular fibrillation; EPS, electrophysiological study.

**Table 2:** Clinical profile of SQTS 1 versus SQTS 2-6.

Variables: SQTS types Affected gen Numbers of patients = 67	SQTS 1 KCNH2 n = 36	SQTS2 -SQTS6 n = 29	p-value
*Gender, n (%)*	male = 20 (50) female = 16 (50)	male = 15 (51) female = 14 (49)	0.9539 0.9539
*Demographics*			
*Age*	34±17	18±17	0.001
*Symptoms, n (%)*			
*Syncope*	9 (22)	3 (9)	0.1222
*Palpitation*	4 (13)	5 (14)	0.4973
*SCD a.a.*	5 (16)	7 (20)	0.3457
*Atrial flutter*	1 (3)	1 (3)	1.0000
*Atrial fibrillation*	6 (19)	7 (20)	0.5397
*nsVT*	1 (3)	1 (3)	1.0000
*Asymptomatic*	17 (47)	8 (26)	0.1735
*ECG Data, mean (n) ± SD*			
*QTc (ms)*	313 ± 31	317±32	0.643
*Medical treatment, n (%)*			
*Yes*	23 (72)	11 (31)	0.0668
*ICD-Implantation, n (%)*			
*Yes*	15 (47)	8 (23)	0.3580
*EPS, n (%)*	2 (85)	0 (20)	0.5016
*Inducible arrhythmia (% from EPS)*	1 (19)	0 (14)	1.0000
*Follow-up time, mean (days)*	2447±2588	2988±1932	0.583
*Events at follow-up, n (%)*	10 (31)	5 (12)	0.3838
*nsVT/VT*	4 (13)	1 (3)	0.3703
*Aborted SCD (VF)*	2 (6)	1 (3)	1.0000
*Death*	0 (0)	1 (3)	0.4462
*Atrial fibrillation*	3 (9)	2 (6)	0.6639
*Syncope*	0 (0)	0 (0)	1.0000
*Atrial flutter*	0 (0)	0 (0)	1.0000
*Palpitation*	1 (3)	0 (0)	1.0000

nsVT, non-sustained ventricular tachycardia; VT, ventricular tachycardia; SCD, sudden cardiac death; VF, ventricular fibrillation; EPS, electrophysiological study.

Atrial fibrillation was significantly higher presented in genotype positive patients as compared to genotype negative patients (atrial fibrillation 19% versus 0%; p = 0.0055). Other symptoms such as syncope, palpitation, SCD, and/or atrial flutter did not show a significant difference in both groups (**Table 1**). The median QTc interval (315 ± 32 versus 320 ± 19; p = 0.302) as well as treatment modality (medical and/or ICD implantation) shows no significant difference in both groups (medical treatment 51% versus 44%; p = 0.6338; ICD implantation 34% versus 35%; p = 0.9523). By comparing the age at the time of diagnosis between patients with genotype positive and negative origin we documented differences without significant meanings (27 ± 19 versus 29 ± 15; p = 0.486). As expected, we documented a significant difference in the male sex (genotype positive 52% versus genotype negative 84%; p = 0.0016) but also in the female sex (genotype positive 45% versus genotype negative 14%; p = 0.0016) (**Table 1**).

### Genetics and Outcome

Since SQTS1 (KCNH2, n = 36) is the most often described SQTS type we compared it with other SQTS types including the CACNB2 (n = 2), CACNA1C (n = 2), CACNA2D1 (n = 1), KCNQ1 (n = 14), KCNJ2 (n = 7), SLC22A5 (n = 2), SCN5A (n = 1), and SLC4A3 (n = 3) (**Table 2**). Symptoms at admission have not shown a significant difference. The QTc interval in both groups shows no significant differences. The median age, on the other hand, has shown a significant difference (p = 0.001). Campuzano et al. (2019) had been discussing the classification of the variants concerning SQTS. They classified the variants according to their pathogenicity following ACMG/AMP recommendations. To avoid misinterpretation of the diagnosis and since only 28.12% of reported variants have a conclusive lethal role in SQTS they recommend to analysis only three genes (*KCNH2*, *KCNQ1*, and *KCNJ2*). This analysis should be performed in all clinical suspected cases of SQTS – other genes such as *CACNA1C*, *CACNA2D1*, and *CACNB2B* should not be analysed. All in all, they recommended an accurate clinical assessment in order to acquire a conclusive SQTS diagnosis before genetic analysis. Due to the lack of information we do not know sufficiently which variants had been analyzed. Of note no functional studies in human cardiomyocytes in other SQTS forms except SQTS type 1 have been established (El-Battrawy et al., 2018a).

Events at follow-up (VT, VF, SCD, AF, atrial flutter, syncope, and palpitation) as outcome data have been compared. No significant difference has been documented (22% versus 9%; p = 0.1195). Even more, data were comparable between single follow-up events such as VT, aborted SCD, death, and other events such as atrial fibrillation, syncope, atrial flutter, and palpitation (**Table 2** and **Table S1**).

The Kaplan-Meier-Curve presents no significant difference of age between genotype positive and genotype negative patients, **Figure S1**.

### Comparison of Males and Females Are Based on the Origin of SQTS

Finally, we compared 67 genotype positive patients with 43 genotype negative patients. The outcome was similar in both groups. Even more, after comparison 33 female SQTS patients with genotype positive origin with 6 genotype negative patients, no differences were found (**Table 3**).

**Table 3:** Comparison between patients with/without confirmed gene mutation and comparison between male and female with/without confirmed gene mutation.

	Confirmed gen	No mutation found	p-Value	Confirmed gen	No mutation found	p-Value	Confirmed gen	No mutation found	p- Value
	All N = 67 Male N = 35 Female N = 30 Unknown N = 2	All N = 43 Male N = 36 Female N = 6 Unknown N = 1		Male = 35	Male = 36		Female = 33	Female = 6	
*Events at follow up*	15 (22)	4 (9)	0.1195	5 (14)	3 (8)	0.7312	10 (30)	1 (17)	1.0000
*Events (nsVT/VT and death and aborted sudden cardiac death) at follow-up, (%)*	9 (13)	4 (9)	0.2554	3 (9)	3 (8)	1.0000	6 (18)	1 (17)	1.0000
*nsVT/VT*	5 (7)	3 (7)	1.0000	0 (0)	2 (6)	0.4932	5 (15)	1 (17)	1.0000
*Death*	1 (2)	1 (2)	1.0000	0 (0)	1 (3)	0.4932	1 (3)	0 (0)	1.0000
*Aborted SCD*	3 (4)	0 (0)	0.5194	3 (9)	0 (0)	0.1093	0 (0)	0 (0)	1.0000
*Arrhythmic event (Palpitations/Syncope/Atrial fibrillation/Atrial flutter)*	6 (9)	0 (0)	0.0796	2 (6)	0 (0)	0.2323	4 (12)	0 (0)	1.0000
*Palpitations*	1 (2)	0 (0)	1.0000	1 (3)	0 (0)	0.4847	0 (0)	0 (0)	1.0000
*Syncope*	0 (0)	0 (0)	1.0000	0 (0)	0 (0)	1.0000	0 (0)	0 (0)	1.0000
*Atrial fibrillation*	5 (7)	0 (0)	0.1543	1 (3)	0 (0)	0.4857	4 (12)	0 (0)	1.0000
*Atrial flutter*	0 (0)	0 (0)	1.0000	0 (0)	0 (0)	1.0000	0 (0)	0 (0)	1.0000

nsVT, non-sustained ventricular tachycardia; VT, ventricular tachycardia; SCD, sudden cardiac death.

## Discussion

The following conclusions were made: (i) whether the symptoms derive from a genotype positive origin or not, does not make a difference except for atrial fibrillation with a predominance in genotype positive patients; (ii) there is no significant difference between the different genetic profiles and the clinical presentation; (iii) genotype positive patients have no higher risk of events at follow-up than genotype negative patients.

SQTS shows a great variability of phenotypes with up to two third of patients experiencing syncope (Garcia-Elias and Benito, 2018). In around one third the SQTS manifests itself through SCD in follow-up, quite often at young age. Nevertheless, there is a great population that remains asymptomatic despite showing continuous short QT intervals on the ECG (El-Battrawy et al., 2018b).

Other than we expected, the clinical presentation of patients with genotype positive was predominated by atrial fibrillation. The higher presence of atrial fibrillation may be related to main cause for admission to the hospital and looking for the related disease. Olesen et al. (2014) has shown that several genes have been found to be associated with atrial fibrillation in early-onset atrial fibrillation patients. According to the study both shortening and prolongation of atrial action potential enhances atrial fibrillation. The study has shown that the prevalence of rare genetic variants in genes associated with atrial fibrillation in the early-onset lone atrial fibrillation patient was almost increased by a factor 2 compared to the background population. The major pharmacological targets in the treatment of atrial fibrillation are ion channels using antiarrhythmic drugs. Due to the fact that some of the genetic variants are found in some of the drug-targeted ion channels, it might explain the less efficacy and the side effects. Furthermore, it might even be a contraindication for some medications. It has been shown that the number of reported familial sudden cardiac deaths (SCD) are higher in patients with early-onset atrial fibrillation.

Overlap syndromes, like SQTS accompanied with Brugada syndrome (BrS), have been reported. BrS has like SQTS a predominance in the male sex and a higher penetrance of SCD especially in the male sex (El-Battrawy et al., 2018c). BrS on the other hand usually remains asymptomatic (Garcia-Elias and Benito, 2018). If symptomatic it has a similar clinical presentation such as syncope or due to ventricular arrhythmic SCD. Also, the age at admission is slightly higher with BrS which is usually between the third and fourth decade.

Dating back to the first characterisation of SQTS a predominance of SQTS 1 has been observed. Contrary to our expectation, neither symptoms were shown to be predictors for SQTS 1 nor for any other SQTS type.

Events at follow-up are not significantly higher with patients with genotype positive. In the study of Mazzanti et al. (2014) they found that the prevalence of the female sex in relation to the male sex was greater among the genotype positive patients. In our study, we discovered the same phenomenon. Even more, genotype positive status was not identified as a predictor of arrhythmic events.

In our study, we documented a higher accumulation of the female sex compared to the male sex in the group with genotype positive. On closer inspection, we could not identify a preference of either of the types of mutation.

In connection with the previously mentioned studies, we recommend to look closer at young patients with early-onset lone atrial fibrillation, especially with the female sex, and where appropriate a closer inspection of the genes in the electric system of the heart.

## Conclusions

Genotype positive SQTS patients have no higher long-term risk of arrhythmic events and/or SCD than genotype negative patients.

## Study Limitations

To a certain extent limitation in subgroup analysis remain, although we included 110 patients from 29 studies incorporating the original data. The small size of the study may render the significance of the negative results less robust.

Since we only used literature reported cases, our study lacks a certain amount of information. Therefore, our study is vulnerable to various bias, including publication bias and measurement bias.

The limitations were exacerbated by the heterogeneous treatment approach based on local centre decision. It is excluded that in some studies SQTS related genes were not screened according to the same protocol of our cohort. Therefore, bias are not excluded.

## Data Availability Statement

All datasets generated for this study are included in the article/**Supplementary Material**.

## Author Contributions

All authors listed have made substantial, direct, and intellectual contribution to the work and approved it for publication.

## Conflict of Interest

The authors declare that the research was conducted in the absence of any commercial or financial relationships that could be construed as a potential conflict of interest.

## Footnotes

1 Data bases include PubMed, Web of Science, Cochrane Library and Cinahl.

## Supplementary Material

Table S1 Overview of included short QT (SQT) types.
