# Supplementary material for: Differences in Short QT Syndrome Subtypes: A Systematic Literature Review and Pooled Analysis
Source: Front Genet. 2020 Jan 17;10:1312. doi: 10.3389/fgene.2019.01312 (PMC6979065; doi:10.3389/fgene.2019.01312)
Supplement: Table S1 — Overview of included short QT (SQT) types. [file Table_1.docx]

| **Study** |  | **El Battrawy 2018** | **Frea**  **2015** | **Hong 2004** | **Priori**  **2005** | **Moreno**  **2015** | **Hattori**  **2011** |
| --- | --- | --- | --- | --- | --- | --- | --- |
| **Overall** | **Overall = 114** | **N = 12** | **N = 15** | **N = 3** | **N =2** | **N = 1** | **N = 1** |
| **Gender, n (%)** | **Unknown= 3 (1)** |  |  |  |  |  |  |
| **Male** | **72 (63)** | 5 (42) | 9 (60) | 1 (33) | 1 (50) | 1 (100) | 0 (0) |
| **Female** | **39 (34)** | 7 (58) | 6 (40) | 2 (67) | 1 (50) | 0 (0) | 1 (100) |
| **Demographics** |  |  |  |  |  |  |  |
| Age, mean | **28 ± 18** | 38 ± 19 | 30 ± 15 | 30 ± 15 | 20 ± 15 | 23 | 8 |
| **Symptoms** |  |  |  |  |  |  |  |
| Syncope | **21 (18)** | 3 (25) | 3 (20) | 0 (0) | 1 (50) | 0 (0) | 0 (0) |
| Palpitation | **10 (9)** | 5 (42) | 3 (20) | 1 (33) | 1 (50) | 0 (0) | 0 (0) |
| Sudden cardiac death at admission | **26 (23)** | 2 (17) | 1 (7) | 0 (0) | 0 (0) | 0 (0) | 0 (0) |
| Atrial flutter | **2 (2)** | 1 (8) | 0 (0) | 0 (0) | 0 (0) | 0 (0) | 0 (0) |
| Atrial fibrillation | **13 (11)** | 4 (33) | 1 (7) | 2 (67) | 0 (0) | 0 (0) | 1 (100) |
| nsVT | **2 (2)** | 0 (0) | 0 (0) | 0 (0) | 0 (0) | 0 (0) | 0 (0) |
| asymptomatic | **45 (39)** | 2 (17) | 8 (53) | 1 (33) | 1 (50) | 1 (100) | 0 (0) |
|  |  |  |  |  |  |  |  |
| **ECG Data, mean (n)** |  |  |  |  |  |  |  |
| QTc (ms**)** | **318± 28** | 328± 40 | 322± 24 | 232± 6 | 317,5± 2,5 | 356 | 194 |
|  |  |  |  |  |  |  |  |
| **Medical treatment, n (%)** |  |  |  |  |  |  |  |
| Yes | **53 (46)** | 5 (42) | 11 (73) | 2 (67) | 0 (0) | 0 (0) | - |
|  |  |  |  |  |  |  |  |
| **ICD-Implantation, n (%)** |  |  |  |  |  |  |  |
| yes | **36 (32)** | 5 (42) | 6 (40) | 3 (100) | - | - | - |
|  |  |  |  |  |  |  |  |
| **Genetic screening, n (%)** |  |  |  |  |  |  |  |
| **Confirmed gen** | **67 (59)** | 12 (100) | 10 (67) | 3 (100) | 2 (100) | 1 (100) | 1 (100) |
| KCNH2 (SQT1) | **32 (28)** | 4 (33) | 7 (47) | 3 (100) | 0 (0) | 0 (0) | 0 (0) |
| KCNQ1 (SQT2) | **14 (12)** | 0 (0) | 3 (20) | 0 (0) | 0 (0) | 1 (100) | 0 (0) |
| KCNJ2 (SQT3) | **7 (6)** | 0 (0) | 0 (0) | 0 (0) | 2 (100) | 0 (0) | 1 (100) |
| KCNJ5 | **1 (1)** | 1 (8) | 0 (0) | 0 (0) | 0 (0) | 0 (0) | 0 (0) |
| CaCNA1c (SQT4) | **3 (3)** | 3 (25) | 0 (0) | 0 (0) | 0 (0) | 0 (0) | 0 (0) |
| CaCNB2b (SQT5) | **5 (4)** | 5 (42) | 0 (0) | 0 (0) | 0 (0) | 0 (0) | 0 (0) |
| CACNA2d (SQT6) | **1 (1)** | 0 (0) | 0 (0) | 0 (0) | 0 (0) | 0 (0) | 0 (0) |
| SLC22A5 | **2 (2)** | 0 (0) | 0 (0) | 0 (0) | 0 (0) | 0 (0) | 0 (0) |
| SCL4A3 | **3 (3)** | 0 (0) | 0 (0) | 0 (0) | 0 (0) | 0 (0) | 0 (0) |
| SCNA5 | **1 (1)** | 1 (8) | 0 (0) | 0 (0) | 0 (0) | 0 (0) | 0 (0) |
|  |  |  |  |  |  |  |  |
| **EPU, n (%)** | **20 (18)** | 6 (50) | - | 3 (100) | 0 (0) | 0 (0) | 1 (100) |
| Inducible arrhythmia (% from EPU) | **13 (65)** | 5 (83) | - | 3 (100) | - | - | 1 (100) |
|  |  |  |  |  |  |  |  |
| **Follow-up time, mean (month)** | **72** | 144 | - | - | - | - | - |
|  |  |  |  |  |  |  |  |
| **Events at follow-up, n (%)** | **19 (17)** | 1 (8) | 5 (33) | 3 (100) | - | - | - |
| nsVT / VT | **8 (7)** | 0 (0) | 4 (27) | 0 (0) | - | - | - |
| Aborted SCD (VF) | **3 (3)** | 1 (8) | 0 (0) | 0 (0) | - | - | - |
| Death | **2 (2)** | 0 (0) | 0 (0) | 0 (0) | - | - | - |
| Atrial fibrillation | **5 (4)** | 0 (0) | 1 (7) | 2 (67) | - | - | - |
| Syncope | **0 (0)** | 0 (0) | 0 (0) | 0 (0) | - | - | - |
| Atrial flutter | **0 (0)** | 0 (0) | 0 (0) | 0 (0) | - | - | - |
| Palpitation | **1 (1)** | 0 (0) | 0 (0) | 1 (33) | - | - | - |

| **Study** | **Guistetto 2006** | **Gollob 2010** | **Deo**  **2013** | **Wu**  **2015** | **Roussel**  **2016** | **Guistetto**  **2015** | **Thorsen**  **2017** |
| --- | --- | --- | --- | --- | --- | --- | --- |
| **Overall**  **N = 81** | **N = 26** | **N =1** | **N = 1** | **N = 2** | **N = 2** | **N = 3** | **N = 3** |
| **Gender, n (%)** |  |  |  |  |  |  |  |
| **Male** | 20 (77) | 1 (100) | 1 (100) | 1 (50) | 1 (50) | 1 (33) | 2 (67) |
| **Female** | 6 (23) | 0 (0) | 0 (0) | 1 (50) | 1 (50) | 2 (67) | 1 (33) |
| **Demographics** |  |  |  |  |  |  |  |
| Age, mean | 34 ± 19 | 13 | 11 | 33 ± 13 | 14,5 ± 13,5 | 28 ± 14 | 31 |
| **Symptoms** |  |  |  |  |  |  |  |
| Syncope | 7 (27) | 0 (0) | 0 (0) | 0 (0) | 0 (0) | 0 (0) | 0 (0) |
| Palpitation | 0 (0) | 0 (0) | 0 (0) | 0 (0) | 0 (0) | 0 (0) | 0 (0) |
| Sudden cardiac death at admission | (23) | 1 (100) | 0 (0) | 1 (50) | 1 (50) | 1 (33) | 1 (33) |
| Atrial flutter | 0 (0) | 0 (0) | 0 (0) | 0 (0) | 0 (0) | 0 (0) | 0 (0) |
| Atrial fibrillation | 0 (0) | 0 (0) | 1 (100) | 0 (0) | 0 (0) | 0 (0) | 0 (0) |
| nsVT | 0 (0) | 0 (0) | 0 (0) | 1 (50) | 0 (0) | 0 (0) | 0 (0) |
| asymptomatic | 13 (50) | 0 (0) | 0 (0) | 0 (0) | 1 (50) | 2 (67) | 2 (67) |
|  |  |  |  |  |  |  |  |
| **ECG Data, mean (n)** |  |  |  |  |  |  |  |
| QTc (ms**)** | 308± 17 | 283 | 283 | 305± 5 | 324,5± 15,5 | 332± 23 | 332± 16 |
|  |  |  |  |  |  |  |  |
| **Medical treatment, n (%)** |  |  |  |  |  |  |  |
| Yes | 6 (23) | - | 1 (100) | 0 (0) | - | 3 (100) | 0 (0) |
|  |  |  |  |  |  |  |  |
| **ICD-Implantation, n (%)** |  |  |  |  |  |  |  |
| yes | 1 (8) | - | - | 1 (50) | 1 (50) | 1 (33) | - |
|  |  |  |  |  |  |  |  |
| **Genetic screening, n (%)** |  |  |  |  |  |  |  |
| **Confirmed gen** | 7 (27) | 0 (0) | 1 (100) | 2 (100) | 2 (100) | 3 (100) | 3 (100) |
| KCNH2 (SQT1) | 7 (27) | 0 (0) | 0 (0) | 0 (0) | 0 (0) | 3 (100) | 0 (0) |
| KCNQ1 (SQT2) | 0 (0) | 0 (0) | 0 (0) | 2 (100) | 0 (0) | 0 (0) | 0 (0) |
| KCNJ2 (SQT3) | 0 (0) | 0 (0) | 1 (100) | 0 (0) | 0 (0) | 0 (0) | 0 (0) |
| KCNJ5 | 0 (0) | 0 (0) | 0 (0) | 0 (0) | 0 (0) | 0 (0) | 0 (0) |
| CaCNA1c (SQT4) | 0 (0) | 0 (0) | 0 (0) | 0 (0) | 0 (0) | 0 (0) | 0 (0) |
| CaCNB2b (SQT5) | 0 (0) | 0 (0) | 0 (0) | 0 (0) | 0 (0) | 0 (0) | 0 (0) |
| CACNA2d (SQT6) | 0 (0) | 0 (0) | 0 (0) | 0 (0) | 0 (0) | 0 (0) | 0 (0) |
| SLC22A5 | 0 (0) | 0 (0) | 0 (0) | 0 (0) | 2 (100) | 0 (0) | 0 (0) |
| SCL4A3 | 0 (0) | 0 (0) | 0 (0) | 0 (0) | 0 (0) | 0 (0) | 3 (100) |
| SCNA5 | 0 (0) | 0 (0) | 0 (0) | 0 (0) | 0 (0) | 0 (0) | 0 (0) |
|  |  |  |  |  |  |  |  |
| **EPU, n (%)** | - | - | 0 (0) | 0 (09 | - | 2 (67) | 0 (0) |
| Inducible arrhythmia (% from EPU) | - | - | - | - | - | 0 (0) | - |
|  |  |  |  |  |  |  |  |
| **Follow-up time, mean (month)** | - | - | 0 | 24 | 156 | 10 | - |
|  |  |  |  |  |  |  |  |
| **Events at follow-up, n (%)** | 2 (17) | - | - | 1 (50) | 0 (0) | 2 (67) | - |
| nsVT / VT | 0 (0) | - | - | 0 (0) | 0 (0) | 2 (67) | - |
| Aborted SCD (VF) | 1 (8) | - | - | 1 (50) | 0 (0) | 0 (0) | - |
| Death | 1 (8) | - | - | 0 (0) | 0 (0) | 0 (0) | - |
| Atrial fibrillation | 0 (0) | - | - | 0 (0) | 0 (0) | 0 (0) | - |
| Syncope | 0 (0) | - | - | 0 (0) | 0 (0) | 0 (0) | - |
| Atrial flutter | 0 (0) | - | - | 0 (0) | 0 (0) | 0 (0) | - |
| Palipitation | 0 (0) | - | - | 0 (0) | 0 (0) | 0 (0) | - |

| **Study** | **Mizobuchi**  **2007** | **Itoh**  **2008** | **Brugada**  **2015** | **Suzuki**  **2014** | **Bun**  **2012** | **Harrel**  **2015** |
| --- | --- | --- | --- | --- | --- | --- |
| **Overall**  **N = 114** | **N = 1** | **N = 3** | **N =3** | **N =1** | **N = 1** | **N = 7** |
| **Gender, n (%)** |  |  |  |  |  |  |
| **Male** | 1 (100) | 2 (67) | 1 (33) | 1 (100) | 1 (100) | 4 (57) |
| **Female** | 0 (0) | 1 (33) | 2 (67) | 0 (0) | 0 (0) | 3 (43) |
| **Demographics** |  |  |  |  |  |  |
| Age, mean | 24 | 32 ± 2 | 0 ± 0 | 10 | 28 | 32 ± 19 |
| **Symptoms** |  |  |  |  |  |  |
| Syncope | 1 (100) | 0 (0) | 0 (0) | 0 (0) | 0 (0) | 1 (14) |
| Palpitation | 0 (0) | 0 (0) | 0 (0) | 0 (0) | 0 (0) | 1 (14) |
| Sudden cardiac death at admission | 0 (0) | 0 (0) | 0 (0) | 0 (0) | 1 (100) | 2 (29) |
| Atrial flutter | 0 (0) | 0 (0) | 0 (0) | 0 (0) | 0 (0) | 1 (14) |
| Atrial fibrillation | 0 (0) | 0 (0) | 3 (100) | 0 (0) | 0 (0) | 1 (14) |
| nsVT | 0 (0) | 1 (33) | 0 (0) | 0 (0) | 0 (0) | 0 (0) |
| asymptomatic | 0 (0) | 2 (67) | 0 (0) | 1 (100) | 0 (0) | 3 (43) |
|  |  |  |  |  |  |  |
| **ECG Data, mean (n)** |  |  |  |  |  |  |
| QTc (ms**)** | 314 | 361± 24 | 297± 9 | 283 | 320 | 322± 18 |
|  |  |  |  |  |  |  |
| **Medical treatment, n (%)** |  |  |  |  |  |  |
| Yes | - | 0 (0) | 2 (67) | 1 (100) | 1 (100) | 1 (14) |
|  |  |  |  |  |  |  |
| **ICD-Implantation, n (%)** |  |  |  |  |  |  |
| yes | - | 1 (33) | 2 (67) | 0 (0) | 1 (100) | 2 (29) |
|  |  |  |  |  |  |  |
| **Genetic screening, n (%)** |  |  |  |  |  |  |
| **Confirmed gen** | 0 (0) | 3 (100) | 3 (100) | 1 (100) | 0 (0) | 5 (71) |
| KCNH2 (SQT1) | 0 (0) | 3 (100) | 0 (0) | 1 (100) | 0 (0) | 4 (57) |
| KCNQ1 (SQT2) | 0 (0) | 0 (0) | 3 (100) | 0 (0) | 0 (0) | 1 (14) |
| KCNJ2 (SQT3) | 0 (0) | 0 (0) | 0 (0) | 0 (0) | 0 (0) | 0 (0) |
| KCNJ5 | 0 (0) | 0 (0) | 0 (0) | 0 (0) | 0 (0) | 0 (0) |
| CaCNA1c (SQT4) | 0 (0) | 0 (0) | 0 (0) | 0 (0) | 0 (0) | 0 (0) |
| CaCNB2b (SQT5) | 0 (0) | 0 (0) | 0 (0) | 0 (0) | 0 (0) | 0 (0) |
| CACNA2d (SQT6) | 0 (0) | 0 (0) | 0 (0) | 0 (0) | 0 (0) | 0 (0) |
| SLC22A5 | 0 (0) | 0 (0) | 0 (0) | 0 (0) | 0 (0) | 0 (0) |
| SCL4A3 | 0 (0) | 0 (0) | 0 (0) | 0 (0) | 0 (0) | 0 (0) |
| SCNA5 | 0 (0) | 0 (0) | 0 (0) | 0 (0) | 0 (0) | 0 (0) |
|  |  |  |  |  |  |  |
| **EPU, n (%)** | 1 (100) | 1 (33) | - | 0 (0) | 0 (0) | 1 (14) |
| Inducible arrhythmia (% from EPU) | 1 (100) | 1 (100) | - | - | - | 0 (0) |
|  |  |  |  |  |  |  |
| **Follow-up time, mean (month)** | 18 | - | 96 | - | 6 | - |
|  |  |  |  |  |  |  |
| **Events at follow-up, n (%)** | 0 (0) | - | 2 (67) | - | 0 (0) | - |
| nsVT / VT | 0 (0) | - | 0 (0) | - | 0 (0) | - |
| Aborted SCD (VF) | 0 (0) | - | 0 (0) | - | 0 (0) | - |
| Death | 0 (0) | - | 1 (33) | - | 0 (0) | - |
| Atrial fibrillation | 0 (0) | - | 1 (33) | - | 0 (0) | - |
| Syncope | 0 (0) | - | 0 (0) | - | 0 (0) | - |
| Atrial flutter | 0 (0) | - | 0 (0) | - | 0 (0) | - |
| Palpitation | 0 (0) | - | 0 (0) | - | 0 (0) | - |

| **Study** | **Templin 2011** | **Ambrosini 2011** | **Bellocq 2004** | **Maltret 2014** | **Redpath 2009** | **Efremidis 2009** |
| --- | --- | --- | --- | --- | --- | --- |
| **Overall**  **N = 114** | **N = 1** | **N = 2** | **N = 1** | **N = 1** | **N =1** | **N = 1** |
| **Gender, n (%)** |  |  |  |  |  |  |
| **Male** | 0 (0) | 2 (100) | 1 (100) | 0 (0) | 1 (100) | - |
| **Female** | 1 (100) | 0 (0) | 0 (0) | 1 (100) | 0 (0) | - |
| **Demographics** |  |  |  |  |  |  |
| Age, mean | 17 | 9 ± 0 | 70 | 0 | 22 | 17 |
| **Symptoms** |  |  |  |  |  |  |
| Syncope | 0 (0) | - | 0 (0) | - | 1 (100) | 1 (100) |
| Palpitation | 0 (0) | - | 0 (0) | - | 0 (0) | 0 (0) |
| Sudden cardiac death at admission | 1 (100) | - | 1 (100) | - | 0 (0) | 0 (0) |
| Atrial flutter | 0 (0) | - | 0 (0) | - | 0 (0) | 0 (0) |
| Atrial fibrillation | 0 (0) | - | 0 (0) | - | 0 (0) | 0 (0) |
| nsVT | 0 (0) | - | 0 (0) | - | 0 (0) | 0 (0) |
| asymptomatic | 0 (0) | - | 0 (0) | - | 0 (0) | 0 (0) |
|  |  |  |  |  |  |  |
| **ECG Data, mean (n)** |  |  |  |  |  |  |
| QTc (ms**)** | 329 | 331 | 302 | 279 | 366 | 283 |
|  |  |  |  |  |  |  |
| **Medical treatment, n (%)** |  |  |  |  |  |  |
| Yes | 1 (100) | 0 (0) | 0 (0) | 1 (100) | 0 (0) | 1 (100) |
|  |  |  |  |  |  |  |
| **ICD-Implantation, n (%)** |  |  |  |  |  |  |
| yes | 1 (100) | - | - | - | 1 (100) | 1 (100) |
|  |  |  |  |  |  |  |
| **Genetic screening, n (%)** |  |  |  |  |  |  |
| **Confirmed gen, n (%)** | 1 (100) | 2 (100) | 1 (100) | 1 (100) | 1 (100) | 0 (0) |
| KCNH2 (SQT1) | 0 (0) | 0 (0) | 0 (0) | 0 (0) | 1 (100) | 0 (0) |
| KCNQ1 (SQT2) | 0 (0) | 0 (0) | 1 (100) | 1 (100) | 0 (0) | 0 (0) |
| KCNJ2 (SQT3) | 0 (0) | 2 (100) | 0 (0) | 0 (0) | 0 (0) | 0 (0) |
| KCNJ5 | 0 (0) | 0 (0) | 0 (0) | 0 (0) | 0 (0) | 0 (0) |
| CaCNA1c (SQT4) | 0 (0) | 0 (0) | 0 (0) | 0 (0) | 0 (0) | 0 (0) |
| CaCNB2b (SQT5) | 0 (0) | 0 (0) | 0 (0) | 0 (0) | 0 (0) | 0 (0) |
| CACNA2d (SQT6) | 1 (100) | 0 (0) | 0 (0) | 0 (0) | 0 (0) | 0 (0) |
| SLC22A5 | 0 (0) | 0 (0) | 0 (0) | 0 (0) | 0 (0) | 0 (0) |
| SCL4A3 | 0 (0) | 0 (0) | 0 (0) | 0 (0) | 0 (0) | 0 (0) |
| SCNA5 | 0 (0) | 0 (0) | 0 (0) | 0 (0) | 0 (0) | 0 (0) |
|  |  |  |  |  |  |  |
| **EPU, n (%)** | 1 (100) | - | 1 (100) | - | - | 1 (100) |
| Inducible arrhythmia (% from EPU) | 1 (100) | - | - | - | - | 0 (0) |
|  |  |  |  |  |  |  |
| **Follow-up time, mean (month)** | 24 | - | 36 | 54 | - | 12 |
|  |  |  |  |  |  |  |
| **Events at follow-up, n (%)** | 1 (100) | - | - | 0 (0) | - | - |
| nsVT / VT | 1 (100) | - | - | 0 (0) | - | - |
| Aborted SCD (VF) | 0 (0) | - | - | 0 (0) | - | - |
| Death | 0 (0) | - | - | 0 (0) | - | - |
| Atrial fibrillation | 0 (0) | - | - | 0 (0) | - | - |
| Syncope | 0 (0) | - | - | 0 (0) | - | - |
| Atrial flutter | 0 (0) | - | - | 0 (0) | - | - |
| Palpitation | 0 (0) | - | - | 0 (0) | - | - |

| **Study** | **Righi 2015** | **Mazzanti 2017** | **Brugada 2004** | **Portugal 2014** |
| --- | --- | --- | --- | --- |
| **Overall**  **N = 114** | **N = 1** | **N = 15** | **N = 2** | **N= 1** |
| **Gender, n (%)** |  |  |  |  |
| **Male** | 0 (0) | 13 (87) | 2 (100) | 1 (100) |
| **Female** | 1 (100) | 2 (13) | 0 (0) | 0 (0) |
| **Demographics** |  |  |  |  |
| Age, mean | 0 | 25 ± 9 | 35,5 ± 15,5 | 52 |
| **Symptoms** |  |  |  |  |
| Syncope | - | 2 (13) | 0 (0) | 1 (100) |
| Palpitation | - | 0 (0) | 0 (0) |  |
| Sudden cardiac death at admission | - | 6 (40) | 1 (50) |  |
| Atrial flutter | - | 0 (0) | 0 (0) |  |
| Atrial fibrillation | - | 0 (0) | 0 (0) |  |
| nsVT | - | 0 (0) | 0 (0) |  |
| asymptomatic | - | 7 (47) | 1 (50) |  |
|  |  |  |  |  |
| **ECG Data, mean (n)** |  |  |  |  |
| QTc (ms**)** | 310 | 332± 12 | 290± 2,5 | 327 |
|  |  |  |  |  |
| **Medical treatment, n (%)** |  |  |  |  |
| Yes | 1 (100) | 15 (100) | - | 1 (100) |
|  |  |  |  |  |
| **ICD-Implantation, n (%)** |  |  |  |  |
| yes | 0 (0) | 7 (47) | 1 (50) | 1 (100) |
|  |  |  |  |  |
| **Genetic screening, n (%)** |  |  |  |  |
| **Confirmed gen, n (%)** | 0 (0) | 2 (100) | 0 (0) | 0 (0) |
| KCNH2 (SQT1) | 0 (0) | 0 (0) | 0 (0) | 0 (0) |
| KCNQ1 (SQT2) | 1 (100) | 1 (50) | 0 (0) | 0 (0) |
| KCNJ2 (SQT3) | 0 (0) | 1 (50) | 0 (0) | 0 (0) |
| KCNJ5 | 0 (0) | 0 (0) | 0 (0) | 0 (0) |
| CaCNA1c (SQT4) | 0 (0) | 0 (0) | 0 (0) | 0 (0) |
| CaCNB2b (SQT5) | 0 (0) | 0 (0) | 0 (0) | 0 (0) |
| CACNA2d (SQT6) | 0 (0) | 0 (0) | 0 (0) | 0 (0) |
| SLC22A5 | 0 (0) | 0 (0) | 0 (0) | 0 (0) |
| SCL4A3 | 0 (0) | 0 (0) | 0 (0) | 0 (0) |
| SCNA5 | 0 (0) | 0 (0) | 0 (0) | 0 (0) |
|  |  |  |  |  |
| **EPU, n (%)** | 1 (0) | - | 1 (50) | 1 (100) |
| Inducible arrhythmia (% from EPU) | 0 (0) | - | 1 (100) | 1 (100) |
|  |  |  |  |  |
| **Follow-up time, mean (month)** | 228 | - | - | 13 |
|  |  |  |  |  |
| **Events at follow-up, n (%)** | 1 (100) | - | 0 (0) | 1 (100) |
| nsVT / VT | 0 (0) | - | 0 (0) | 1 (100) |
| Aborted SCD (VF) | 0 (0) | - | 0 (0) | 0 (0) |
| Death | 0 (0) | - | 0 (0) | 0 (0) |
| Atrial fibrillation | 1 (100) | - | 0 (0) | 0 (0) |
| Syncope | 0 (0) | - | 0 (0) | 0 (0) |
| Atrial flutter | 0 (0) | - | 0 (0) | 0 (0) |
| Palpitation | 0 (0) | - | 0 (0) | 0 (0) |

Abbreviations:

nsVT – non-sustained ventricular tachycardia

VT – ventricular tachycardia

SCD – sudden cardiac death

VF – ventricular fibrillation
